# A List of Cases Discharged from the Surgical Wards of the Pennsylvania Hospital, during the Month of November, 1843

**Published:** 1843-12-30

**Authors:** Ellerslie Wallace

**Affiliations:** Resident Surgeon


### A LIST OF CASES

### DISCHARGED FROM THE SURGICAL WARDS OF THE PENNSYLVANIA HOSPITAL,

*During the month of November*, 1843.

[Reported by ELLERSLIE WALLACE, M. D., Resident Surgeon.]

(Dr. RANDOLPH Attending Surgeon.)

*The cases marked * are referred to below the list.*

| | |
|---|---|
| Luxation of humerus into axilla, | 1 |
| *Luxation of elbow, both bones, | 1 |
| *Fracture of femur, | 1 |
| *Fracture of surgical neck of humerus, | 1 |
| Fracture of outer condyle of humerus, | 1 |
| *Compound fracture of os frontis, with fracture of os ethmoides, ossa nasi, and os maxillare inferiors, | 1 |
| Compound fracture of tibia, | 1 |
| Contusions, | 4 |
| *Sprained ankle, | 1 |
| Incised wounds, | 3 |
| Lacerated wounds, | 3 |
| Gun-shot wound, | 1 |
| Fistula in perineo, | 1 |
| Abscess in perineo, | 1 |
| Ulcers, | 2 |
| Onychia, | 1 |
| Caries of metatarsal bones, | 1 |
| Cancer of lip, | 1 |
| Carcinoma, (extirpated by Dr. Randolph,) | 1 |
| Scrofula, | 1 |
| Lichen, | 1 |
| Impetigo, | 1 |
| Coxalgia, | 1 |
| Periostitis, | 1 |
| Sclerotitis, | 1 |
| Keratitis, | 1 |
| Phymosis, | 2 |
| Gonorrhœa, | 1 |
| Gonorrhœa, with secondary syphilitic eruption, | 1 |
| Syphilis, primary, | 1 |
| Syphilis, secondary, | 2 |
| Aneurism of the external iliac artery,† | 1 |
| *A patient trephined by Dr. Peace, | 1 |

#### LUXATION OF ELBOW.

This accident occurred in a boy about 7 years old. He had thrust out his arm to save himself from falling, and the weight of his body was received upon the palmar surface of his hand near the wrist joint. He came into the ward about fifteen minutes after the fall, and before any swelling had come on which could obscure the diagnosis of the injury. The bones of the fore-arm were found to be dislocated backward and slightly outward upon the humerus. The forearm was in an extended position, the hand was supinated, and pronation could not be fully effected. The heads of the ulna and radius could be seen projecting posteriorly against the skin; and the rotation of the head of the radius could be distinctly seen and felt in its unnatural position. The condyles of the humerus could also be felt on the anterior aspect of the joint.

The reduction was readily accomplished, thus: an assistant grasped the arm firmly, and maintained it fixed, at the same time making pressure on the upper part of the fore-arm to throw it off the condyles. The forearm was now flexed slowly and firmly; moderate extension being at the same time made from the wrist. A distinct snap announced the moment of reduction. The head of the radius being disposed to rise again upon the condyle, was kept in situ by a small compress, secured by a moderately tight roller. The limb was now placed upon an angular splint and supported in a sling. Very slight inflammation followed, and the swelling also was of small extent. The joint was kept at perfect rest for eighteen days; passive motion was then commenced, with local frictions and hot bathing; and the boy was discharged in twenty-eight days from the receipt of the injury, with almost perfect motion of the part.

#### FRACTURE OF OS FEMORIS.

These injuries are generally treated in the Hospital by Desault's Apparatus, with Dr. Physick's modification. The patients are usually kept in the splints for about two months, and are then allowed to move about with the aid of crutches; the bone being supported meanwhile by pasteboard splints, moulded and bandaged to the thigh. This treatment being continued for from two to three weeks, the bone is sufficiently firm, and the motions of the limb so far recovered, that they are in a condition to be discharged.

In the case referred to above, the patient, a lad about eighteen years of age, was kept at rest for eight weeks, when he was discharged at his own request, being yet unable to walk without a crutch. The fracture had occurred at the junction of the upper and middle third of the femur.

Three days after his discharge he was again brought into the ward, having refractured the bone apparently at the seat of the original injury, by falling on the pavement with the thigh under him. The same apparatus was again applied, and he was kept in the splints for nine weeks, and then allowed to

† This case will be noticed hereafter.

move about on crutches. The correctness of Dupuytren's statement, that a fracture of the provisional callus requires as long a time for consolidation to take place as the ordinary fractures of bones, was in this case beautifully exemplified. In seventy-four days from the date of the second fracture he was discharged, being able to walk well with the assistance of a cane. A very careful measurement showed the shortening to be one-third of an inch.

### FRACTURE OF SURGICAL NECK OF HUMERUS.

The patient was a man 52 years of age. Upon entering the hospital he stated that his shoulder was dislocated, and that a young physician in the northern part of the city, to whom he applied within an hour after the injury, had, with the assistance of *eight strong men*, vainly endeavoured to reduce the luxation by pulling his arm—fortunately not from his body—although the extension was kept up until the Doctor had exhausted both his skill and his patience. He was then advised to come to the hospital, as he "would there find more powerful means of reduction."

When he entered the ward, about twelve hours after the accident, the arm was observed to be swollen at its upper part, but no tumour could be found in the axilla, nor was the rotundity of the shoulder lost; the elbow also was near to the side, and motion was not impeded, as in luxation. The elbow being now rotated, the head of the os humeri could be felt in its natural position, and nearly motionless. This motion also discovered crepitus, and the point of fracture was ascertained to be just above the insertion of the pectoralis major, &c. A wedge-shaped pad, with the large end uppermost, was placed between the arm and the side, high up in the axilla, and the forearm supported in a sling, allowing the elbow to make extension by its own weight. No other dressing could be applied at first, as there were great swelling and violent inflammation, consequent partly upon the injury, but more upon the torture to which the poor fellow had been subjected. Free local depletion was resorted to, and after the lapse of six days the violent symptoms were sufficiently overcome to admit of the usual apparatus being used. A roller was applied from the ends of the fingers to the axilla, a pad was placed as before mentioned, a single splint was laid on the outside of the arm, and, the weight of the elbow being allowed to make extension, a roller was passed around the arm and body of the patient, confining the limb perfectly at rest, and the forearm was supported in a sling. After being under treatment fourteen days his business obliged him to leave the hospital; but when we last heard from him, the fracture was perfectly consolidated without deformity.

### COMPOUND FRACTURE OF OS FRONTIS, &c.

The subject of this injury was a negro, aged 24 years, who had received a blow on the forehead just above the nose, from a cane, which was so severe as to knock him down; but he could not tell the position in which he had fallen, as he was somewhat intoxicated when he came in, which was about 10 o'clock, P. M.

A compound and somewhat comminuted fracture of the external wall of the frontal sinus was found where the blow had been inflicted, but whether the internal table was also fractured, could not be certainly ascertained. He had lost a good deal of blood from the wound, but a compress of dry lint soon arrested the hemorrhage. The bones of the nose were also broken, and the lower jaw was fractured near the symphysis, and on each side of it; the fragments were placed in apposition, and Barton's bandage was applied to retain them. When the intoxication passed off he was observed to be labouring under slight symptoms of concussion, which continued until about sixteen hours after his entrance, when he had a violent convulsion. He was bled f℥xxij., the convulsion passing away, and his mind returning under the depletion.

Evidences of meningitis soon appearing, he was again depleted; cups were freely applied to the back of the neck and between the shoulders; cold water was applied to the head, and revulsives were used to the extremities. He was also actively purged, and his diet was restricted to gruels. Notwithstanding all endeavours he continued to become worse, with delirium, partial convulsions, strabismus and coma, and he died in five days from the time of his admission. There had been no paralysis of either side, and the convulsive spasms had affected both sides about equally.

*Autopsy made fifteen hours after Death.*

External wound nearly healed. There was ecchymosis in the substance of the scalp, about two inches above the left eye; the scalp elsewhere was not unusually vascular. The occipito-frontalis muscle, in the neighbourhood of the injury, was infiltrated with pus and blood. The external wall of the frontal sinus was comminuted and somewhat depressed, for a space equal in size to a dollar. The internal table opposite was broken in a stellated manner, and driven in upon the dura mater; the median line of the skull passed through the centre of the fracture, which was about the size of a quarter of a dollar. At this point, between the skull and dura mater, there was a small effusion of blood. Upon laying open the cavity of the arachnoid, a considerable quantity of sero-purulent fluid escaped. There was a large effusion of blood in the pouches, formed by the junction of the falx major with the tentorium—the amount being about the same on each side; that part of the arachnoid covering the superior convolutions of the brain, was coated with a layer of firm yellowish lymph, and there was abundant sero-purulent effusion between the arachnoid and pia mater, over a corresponding extent. At the base of the brain, and in the ventricles, the arachnoid was healthy. The pia mater was intensely injected, but not adherent to the brain, excepting at the seat of the fracture, where the brain was found to be ecchymosed and softened for a space about as large as a hickory nut; and at this place there was a large and firm coagulum of lymph between the pia mater and arachnoid. The brain was firm, and healthy in appearance elsewhere. The crista galli of the ethmoid bone was fractured at its base, and moveable, but not driven up towards the brain.

### SPRAINED ANKLE.

The mode of treatment generally adopted in cases of this kind, is to place the patient on his back, and after securing the foot and leg in a fracture box, to elevate the limb upon a single inclined plane, apply cold applications, leeches, &c., as the case may demand. Purgatives are also prescribed, and low diet is likewise ordered. After the early and active symptoms pass off, the joint is still kept at perfect rest in the fracture box, until all pain and tenderness have disappeared, which takes place in from three to five weeks in a majority of cases. The limb being then enveloped in a roller, the patient is ordered to move about, and a perfect cure is thus usually effected.

The patient above noticed came into the hospital ten days after the injury had been received. The soreness about the joint was excessive, and he was unable to put the foot to the ground. Upon inquiry, he said that, contrary to the orders of his surgeon, he had left his bed a few days before, and upon going into the yard had fallen upon the ice, and with the effect of aggravating all the bad symptoms. The treatment above referred to was adopted, and in eighteen days we removed the fracture box, and applying a soap plaster and a roller to the part, the man was able to walk about the ward without pain. In twenty-two days from the time of his entrance, he was discharged, cured.

The subject of the operation of trephining was a man who had been a sailor in the service of the United States. About fifteen months previously he had fallen against a gun-carriage, whereby he had received a depressed fracture of the skull, commencing about an inch above the right superciliary ridge, and extending upward and backward for about the space of an inch. The statement given by the patient was, that a piece of bone had been removed immediately after the receipt of the injury, and that he had remained insensible for a short time; that he had been on the sick list for three weeks, and then had returned to his duty; but that in a few weeks his powers of mind had failed him, so that he could not remember names, terms, orders, &c. About the same time his power of locomotion also failed, so that he staggered and fell about the deck. He remained nearly in this condition until the time of his admission into the hospital. While here, before the operation, he was observed to be subject to paroxysms of increased mental alienation, amounting at times to idiocy or insanity. His physical condition was but slightly variable. When he was brought into the amphitheatre, Dr. Peace stated to the class that he considered the case almost hopeless, but that he deemed it proper to give the man his only chance of recovery by removing the depressed portion of bone; and referred to a case of Mr. Cline's, where the patient had been injured thirteen months before, and in which the operation was attended with complete success.*

After the removal of the depressed portion of bone by the crown of the trephine, the flaps of the scalp were replaced, the edges of the wound lightly supported by a strip of adhesive plaster, and a piece of dry lint laid over it. Union took place by granulation; but the condition of the man was in no wise improved, and he was discharged as incurable.

* Sir A. Cooper's Lectures, by Lee. Lect. xvii.

